# A Rare Presentation of an Adverse Reaction to Minocycline

**DOI:** 10.7759/cureus.69160

**Published:** 2024-09-11

**Authors:** Francisca Manoel, José Maria Lupi, Isabel V Coelho, Andreia Ferrão, Joana Gaspar

**Affiliations:** 1 Pediatrics, Hospital do Espírito Santo de Évora, Évora, PRT; 2 Allergy and Immunology, Hospital do Espírito Santo de Évora, Évora, PRT

**Keywords:** drug-induced eosinophilia, drug-induced pneumonitis, drug-induced urticarial rash, drug reaction with eosinophilia and systemic symptoms (dress) syndrome, minocycline, pediatric drug reaction

## Abstract

Drug reaction with eosinophilia and systemic symptoms (DRESS) is a rare, severe adverse drug reaction, usually associated with antibiotics and anticonvulsants. A 17-year-old girl with maculopapular rash, arthralgia, fever, and facial edema (plus eosinophilia and hepatitis) repeatedly goes to the emergency department, initially omitting having started minocycline three weeks before symptom onset. Diagnosis of serum-like sickness was first established, minocycline was suspended, and a short course of corticosteroid therapy was started. However, the fast taper of corticotherapy resulted in the reappearance of previous symptoms, as well as renal dysfunction and respiratory distress. Chest CT showed interstitial pneumonitis. With these findings, the final diagnosis of DRESS was made, and the re-adjustment of corticoid therapy resulted in symptom improvement. This case highlights the diagnostic and treatment challenges of DRESS and the importance of an all-around approach to the patient.

## Introduction

Drug reaction with eosinophilia and systemic symptoms (DRESS) is a rare and potentially fatal adverse drug reaction [1,2]. The incidence of DRESS is around 1:100.000 cases per year in the general population with an overall mortality described as high as 10%. Both these values appear to be lower in the pediatric population but their real numbers are still unknown [1,3]. Antibiotics, such as beta-lactams and macrolides, and aromatic anticonvulsants, such as carbamazepine and phenytoin, are the most common triggers of DRESS [1].

The symptoms appear two to six weeks after starting a new medication and usually disappear more than three weeks after discontinuation of the culprit drug. The hallmarks of DRESS are fever, lymphadenopathy, skin eruption, facial edema, eosinophilia, and organ dysfunction [4]. The liver is the most frequently affected organ but renal, gastrointestinal, cardiac, pulmonary, thyroid, and brain involvement can also occur. In children, the lungs are less commonly affected but may be associated with more severe disease and higher mortality [1,5].

We present a case of severe DRESS caused by minocycline, which evolved with pulmonary, hepatic, and renal involvement. With this report, we aim to emphasize the importance of adequate history taking, the variety of possible symptoms that may not all manifest at the same time, the relevance of a close follow-up, and the need for slow steroid reduction, as well as the consequences of an overly fast taper.

## Case presentation

**Figure 1:**
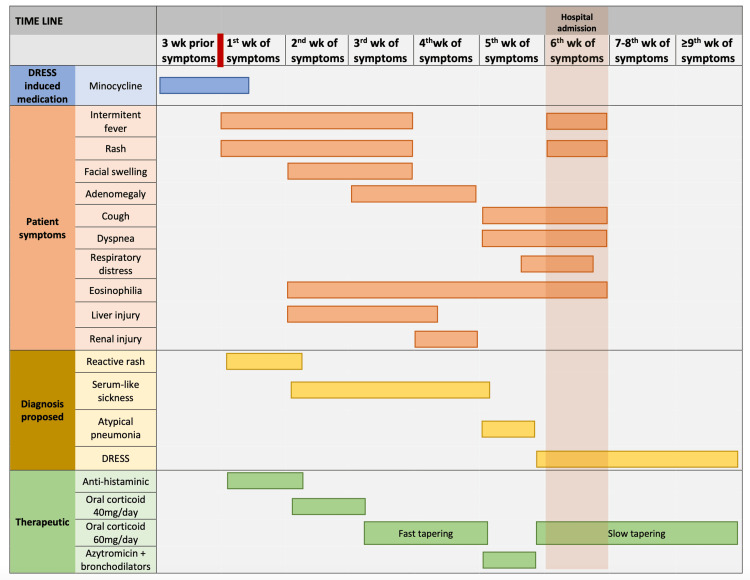
Patient timeline

A 17-year-old Caucasian girl, with no relevant medical history other than recurrent ear infections and acne, presented to the pediatric emergency department of a level 2 hospital with a three-day history of a disseminated maculopapular rash, with periods of urticaria lesions starting in the dorsal region, wrist and shoulder arthralgia, and two days of sensation of chest oppression and intermittent fever. A timeline is shown in Figure 1 denoting symptoms, investigation, and therapeutic approach.

**Figure 2:**
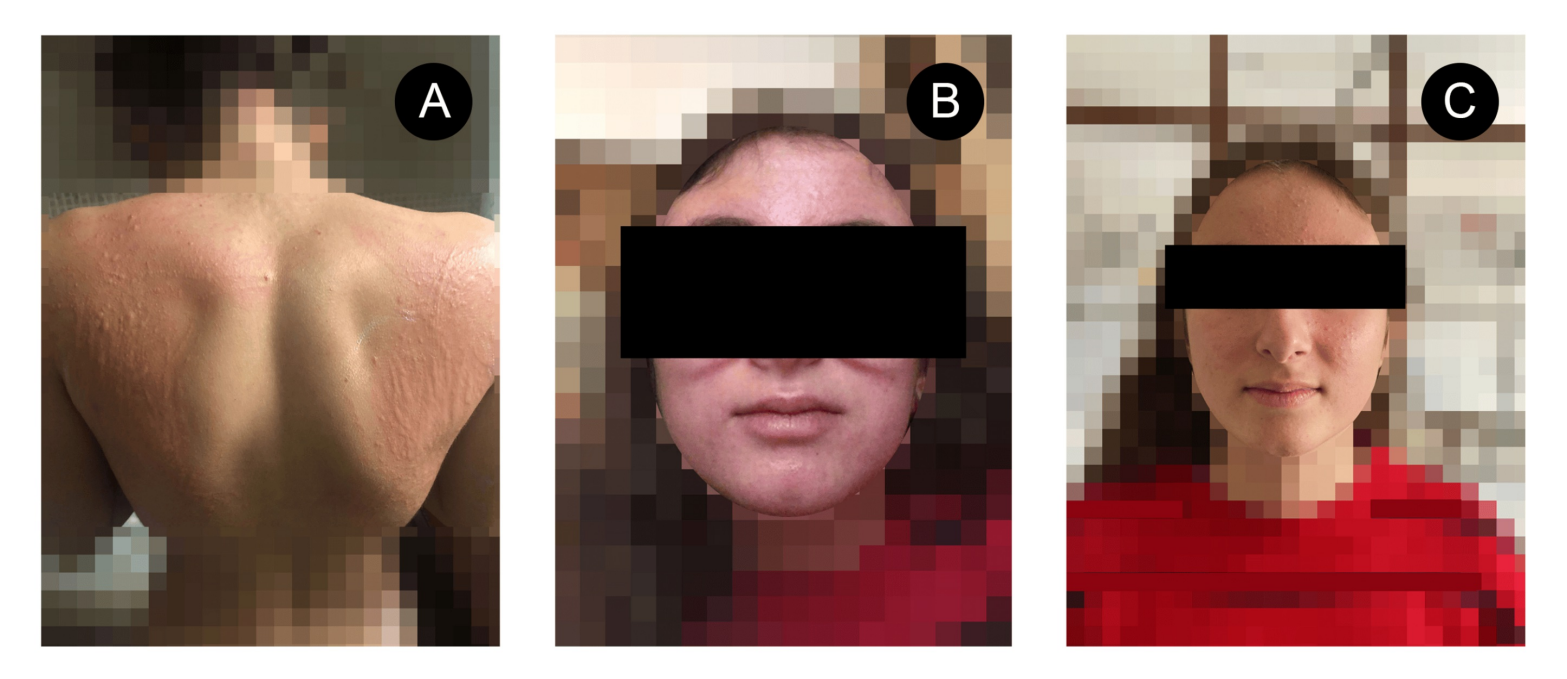
A. maculopapular rash with dermatographism; B. facial edema during symptomatic phase; C. improvement of facial edema after treatment

She was initially discharged with oral antihistamines but failed to improve, returning several times the following week due to symptom worsening, with the emergence of dermatographism (Figure 2A) and facial edema (Figure 2B). On her third visit, blood tests were performed, which revealed marked eosinophilia (14%), without anemia or low platelets, elevated liver enzymes (alanine transaminase (ALT) 132 U/L, aspartate transaminase (AST) 55U/L), and negative immunoglobulin M (IgM) Epstein-Barr virus (EBV) viral capsid antigen (VCA). After further inquiries, it was revealed the patient had begun treatment with minocycline 100 mg once daily three weeks prior to treatment for severe acne. A presumptive diagnosis of serum-like sickness was established at the time, and the patient suspended minocycline and began corticotherapy with prednisolone 40 mg/day.

Two days later, the skin lesions generalized with characteristics of morbilliform eruption, pruritic, without mucosal, hand or feet involvement; with persistence of intermittent fever, two spikes a day (maximum 39ºC), for the last nine days. At this time multiple cervical, occipital, axillary, and inguinal lymphadenopathies were palpable. Analytically, a decrease in eosinophilia to 6% and persistence of elevated liver enzymes (ALT 159 U/L; AST 59 U/L) with only mild elevation of inflammation markers (C-reactive protein (CRP) 1.5 mg/dL; erythrocyte sedimentation rate (ESR) 18 mm/h) was documented. In accordance with the immunoallergology department, prednisolone was increased to 60 mg/day with a taper plan lasting 23 days. Symptom remission was achieved after four days. 

Three weeks later (at the time with 5 mg prednisolone), the pruritic skin lesions and intermittent fever returned, accompanied by a dry cough and dyspnea. A chest radiograph revealed a bilateral interstitial infiltrate. An atypical pneumonia was assumed and azithromycin and bronchodilators were started.

**Figure 3:**
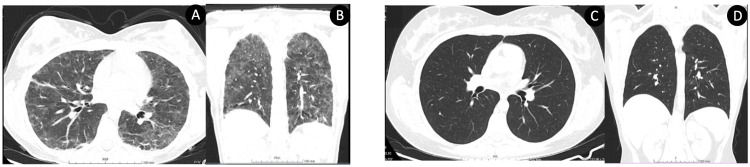
A and B. CT during the symptomatic phase; C and D. CT after treatment CT: computed tomography

After five days, when the medication prescribed produced no effect, a marked worsening of the respiratory status was noted. The patient presented multiple signs of respiratory distress (polypnea, nasal flaring, and difficulty speaking), with a peripheral O2 saturation of 93% and a pulmonary auscultation with decreased murmur, without rales or wheezing. A chest CT showed a ground glass pattern with diffuse, heterogeneous parenchymal opacities with a peribronchovascular distribution and bilateral pleural effusion, suggestive of interstitial-alveolar disease (Figure 3A, 3B). The blood test revealed an elevation of CRP to 12.1 mg/dL and ESR of 32 mm/h, leucocytosis (12.900/uL), normal count of neutrophils and lymphocytes, eosinophilia (19%), thrombocytosis (460.000/uL), and a 1.5-fold increase in serum creatinine (0.66 to 1 mg/dL), with a glomerular filtration rate (GFR) that decreased from 96 to 64 mL/min/1.73 m^2^ and without proteinuria or hematuria. Incidentally, liver enzymes were normal in this evaluation.

After a protracted period of symptoms lasting more than a month and faced with a history of intermittent fever, pruritic rash, lymphadenopathy, facial edema, and eosinophilia, associated with multiorgan dysfunction involving the liver, kidneys, and lungs, a diagnosis of DRESS was finally established and a high dose of prednisolone (60 mg/day) was restarted, with a much slower taper over four months, leading to a sustained clinical, analytical, and radiological improvement, with no relapses (Figure 2C). Supplemental oxygen therapy lasted for five days and hospital admission for six days. Intermittent fatigue and joint/muscle pain persisted for a few weeks but without fever or other accompanying symptoms.

During the hospital stay, acute infectious disease was ruled out, with the exception of the isolation of rhinovirus RNA in respiratory secretions, which was ruled out as a likely cause of the patient’s symptoms. Of note, HAV, HCV, HHV6, CMV, and EBV were negative. Cardiac involvement was excluded due to negative troponin titers and a normal EKG and echocardiogram. Regarding auto-immune markers, anti-nuclear antibodies, anti-DsDNA, anti-CCP IgG, and anti-cardiolipine antibodies were negative/normal.

Follow-up was maintained for a further eight months in an outpatient setting, with normalization of liver enzymes, renal function, and inflammatory markers. A chest CT was repeated four weeks after restarting high-dose prednisolone, revealing a resolution of the ground-glass pattern with no apparent sequelae or scarring (Figure 3C, 3D), which was further corroborated by a normal pulmonary function test.

## Discussion

This case demonstrates how the asynchronous appearance of symptoms and their overlap may complicate the diagnosis of DRESS and delay the initiation of treatment, worsening the prognosis. As described in the literature [3-5], frequent differential diagnoses of acute urticaria, serum-like sickness, viral/atypical pneumonia, and autoimmune disease were all considered. Of note, only after the third visit to the emergency department was the teenager found to have started a new medication three weeks before. This information turned out to be part of the key to diagnosis and treatment, demonstrating that an adequate history taking, with a special focus on personal background and recent initiation of medication, should always be performed, lest a crucial diagnosis may slip unnoticed.

In spite of the typical clinical presentation, which fulfills all of the DRESS RegiSCAR criteria [6], there are some particular points of interest in the clinical course of this case. The initial presentation with wrist and shoulder arthralgia confounded the diagnosis, owing to its little specificity while also comprising part of the typical features of serum-like sickness, being seldom observed in DRESS [3,4]. On the other hand, upon review, the presence of eosinophilia is a specific diagnostic criterion for DRESS and should have led to the clinical suspicion of the syndrome, being typically increased in blood, skin, and involved organs [1]. The frequency of pulmonary involvement in children and adolescents is poorly established in the literature but appears to be rare. In More F et al. [1] and Kim et al. [5], studies conducted only in the pediatric population, pulmonary involvement occurs in 2.6-5% and 20.9%, respectively. In other studies carried out in populations of all ages, lung involvement occurs in 5-33% of cases [7-10]. This involvement may be associated with more severe disease and higher mortality, with dyspnea, cough, and/or pleurisy being the most common presentations [7]. Taweesedt et al. [11] showed that the most common radiologic pulmonary manifestation was pneumonitis (50%), as in this case, followed by acute respiratory distress syndrome (31%), pleural effusion (22.7%), pneumonia, and pulmonary nodules. Allopurinol, abacavir, and especially minocycline are most commonly implicated when lung involvement occurs [1,6,12]. 

In this clinical case, according to the 2011 update of the French causality assessment method [13], minocycline is the very likely culprit. With respect to chronological criteria [13], the onset of symptoms three weeks after initiation of the drug is incredibly consistent with the three to four weeks stipulated in the literature [1,3-5,12]. Furthermore, the persistence of symptoms after discontinuation of minocycline for a substantial period, in probable relation to corticoid fast taper, does not preclude DRESS from consideration. Moreover, according to the semiological criteria [13], the clinical pattern of reaction related to minocycline in the literature is consistent with our case. Other non-drug causes, such as infections like HAV, HCV, HHV6, CMV, and EBV [1,2,5,7,10-13], were also excluded. As we can see in a retrospective study performed by Peyrière et al. [12], the minocycline severe side effects occurred more frequently in younger patients (median age 19.5 years (14-48)). Its usual nonspecific combination of manifestations related to minocycline are peripheral adenopathies in 80%, eosinophilia in 72%, heart abnormalities, in particular, myocarditis and pericarditis in 22%, and eosinophilic pneumopathy in 33% [12].

We believe the hypothesis raised by Peyrière et al. [12], namely, the possibility that DRESS is in fact an aggregation of multiple severe adverse drug reactions with different afflictions and possibly underlying mechanisms depending on the culprit drug, merits consideration. However, due to the rarity of the disease, obtaining a large enough sample size to adequately research this hypothesis will probably prove to be very difficult.

The treatment of pediatric DRESS, due to a lack of controlled trials and prospective studies, remains a challenge. It is known that corticoid therapy should be administered in all patients with moderate to severe DRESS and its tapering should take two to six months [5,7,10,11,14,15]. One of the difficulties in the management of this case was the fact that corticoid tapering had already been tried rapidly before (23 days), with worsening of symptoms at lower doses.

This prolonged tapering is essential to reduce the likelihood of relapses and development of autoimmune sequelae, even upon resolution of clinical manifestations, because these patients are at greater risk of developing an immune reconstitution inflammatory syndrome (IRIS) ranging from hyperinflammation against latent infections (CMV, tuberculosis, herpes zoster/simplex, and sarcoidosis) to the (re)appearance of DRESS manifestations or new autoimmune diseases [7,14,16].

After the final diagnosis was made, the dose was readjusted to therapeutic levels and tapering was performed in several months, according to clinical evolution, as recommended in the literature [1,5,7,10,11,14,15], with no further recurrence of symptoms.

Despite complete clinical, analytical, and radiological resolution, a drug lymphocyte stimulation testing should have been performed, in order to confirm the drug adverse reaction. It is also never excessive to underline the importance of reporting cases such as this one to pharmacovigilance authorities [10].

## Conclusions

DRESS is a rare syndrome with an enormous diversity of clinical presentations, which vary between patients and culprit drugs.

This rare case is a solid example of how a high degree of clinical suspicion, as well as adequate history taking, are needed for the establishment of a prompt and accurate diagnosis. Culprit drug withdrawal and a slow tapering of corticotherapy are imperative for the proper resolution of these cases, improving prognosis. Long-term follow-up is recommended to monitor the relapse of symptoms and aforementioned comorbidities, as well as the progression to other forms of autoimmune diseases.

To finish, we would like to highlight the importance of the general pediatricians/clinicians to recognize this syndrome and its presentation, despite its rarity. After several observations, this final diagnosis was established in the emergency room by a general pediatrician, showing that despite the need for ever-increasing specialization in the medical field, a holistic, multisystem approach to the patient remains an ageless requirement of our profession.
